# Research Trends in the Effect of Caffeine Intake on Fat Oxidation: A Bibliometric and Visual Analysis

**DOI:** 10.3390/nu15204320

**Published:** 2023-10-10

**Authors:** Jorge Gutiérrez-Hellín, Juan Del Coso, Mário C. Espada, Víctor Hernández-Beltrán, Cátia C. Ferreira, David Varillas-Delgado, Nuria Mendoza Laiz, Justin D. Roberts, José M. Gamonales

**Affiliations:** 1 Facultad de Ciencias de la Salud, Universidad Francisco de Vitoria, Ctra. Pozuelo-Majadahonda Km 1.800, 28223 Pozuelo de Alarcón, Spain; david.varillas@ufv.es (D.V.-D.); nuria.mendoza@ufv.es (N.M.L.); or martingamonales@unex.es (J.M.G.); 2 Sport Sciences Research Centre, Universidad Rey Juan Carlos, 28943 Fuenlabrada, Spain; 3 Instituto Politécnico de Setúbal, Escola Superior de Educação, 2914-504 Setúbal, Portugal; mario.espada@ese.ips.pt (M.C.E.); catia.ferreira@ese.ips.pt (C.C.F.); 4 Life Quality Research Centre (CIEQV-Leiria), 2040-413 Rio Maior, Portugal; 5 CIPER, Faculdade de Motricidade Humana, Universidade de Lisboa, 1499-002 Lisboa, Portugal; 6 Facultad Ciencias del Deporte, Universidad de Extremadura, 10005 Cáceres, Spain; vhernandpw@alumnos.unex.es; 7 Cambridge Centre for Sport and Exercise Sciences, School of Psychology and Sport Science, Anglia Ruskin University, Cambridge CB1 1PT, UK; justin.roberts@aru.ac.uk; 8 Programa de Doctorado en Educación y Tecnología, Universidad a Distancia de Madrid, 28400 Collado Villalba, Spain

**Keywords:** bibliometric analysis, coffee, caffeine effects, dietary supplement, fat loss, weight loss

## Abstract

In the last few decades, numerous studies pertaining to research groups worldwide have investigated the effects of oral caffeine intake on fat oxidation at rest, during exercise, and after exercise. However, there is no bibliometric analysis to assess the large volume of scientific output associated with this topic. A bibliometric analysis of this topic may be used by researchers to assess the current scientific interest in the application of caffeine as a nutritional strategy to augment fat oxidation, the journals with more interest in this type of publication, and to draw international collaborations between groups working in the same area. For these reasons, the purpose of this study was to assess the research activity regarding oral caffeine intake and fat oxidation rate in the last few decades by conducting a bibliometric and visual analysis. Relevant publications from 1992 to 2022 were retrieved from the Web of Science (WoS) Core Collection database. Quantitative and qualitative variables were collected, including the number of publications and citations, H-indexes, journals of citation reports, co-authorship, co-citation, and the co-occurrence of keywords. There were 182 total publications, while the number of annual publications is saw-shaped with a modest increase of 11.3% from 2000 to 2009 to 2010 to 2019. The United States was the country with the highest number of publications (24.17% of the total number of articles), followed by the Netherlands (17.03%). According to citation analyses, the average number of citations per document is 130, although there are 21 documents that have received more than 100 citations; the most cited document reached 644 citations. These citation data support the overall relevance of this topic in the fields of nutrition and dietetics and sport sciences that when combined harbored 85.71% of all articles published in the WoS. The most productive author was Westerterp-Plantenga with 16 articles (8.79% of the total number of articles). Nutrients was the journal that published the largest number of articles on this topic (6.59% of the total number of articles). Last, there is a tendency to include keywords such as “performance”, “carbohydrate”, and “ergogenic aid” in the newer articles, while “obesity”, “thermogenic”, and “tea” are the keywords more commonly included in older documents. Although research into the role of caffeine on fat oxidation has existed since the 1970s, our analysis suggests that the scientific output associated with this topic has progressively increased since 1992, demonstrating that this is a nutritional research area with a strong foundational base of scientific evidence. Based on the findings of this bibliometric analysis, future investigation may consider focusing on the effects of sex and tolerance to caffeine to widen the assessment of the effectiveness of oral caffeine intake as a nutritional strategy to augment the use of fat as a fuel, as these terms rarely appear in the studies included in this analysis. Additionally, more translational research is necessary as the studies that investigate the effect of oral caffeine intake in ecologically valid contexts (i.e., exercise training programs for individuals with excessive adiposity) are only a minor part of the studies on this topic.

## 1. Introduction

Caffeine (1,3,7-trimethylxanthine) is an alkaloid belonging to the group of methylxanthines. Caffeine is found naturally in the leaves, fruits, and seeds of various plants (coffee, tea, mate, cocoa, etc.) although it can also be artificially synthesized and added to manufactured products. In fact, there is an extensive market of caffeine-containing products and dietary supplements worldwide that includes dozens of forms of caffeine administration using either natural or artificial sources for caffeine. One of the most common methods of daily caffeine intake is through herbal infusions (mainly coffee), although the most common method of caffeine ingestion may depend on age and country [1]. Coffee infusions contain more caffeine than other beverages and contribute significantly more to overall caffeine consumption that included over 1 billion people worldwide drinking coffee every day and an average of 2.25 billion coffee cups consumed each day, according to the National Coffee Association of the United States [2]. The high presence of caffeine in different foods, beverages, and dietary supplements and the efficacy of this substance to increase stamina have resulted in caffeine being the most widely consumed psychostimulant globally [3]. Along with the high use of caffeine, there is a growing public and scientific interest in identifying the potential health consequences of regular caffeine intake in its different formats [4]. Overall, although caffeine is primarily ingested to enhance physical performance, alertness, and vigilance, long-term caffeine ingestion in moderate doses may pose a beneficial role in reducing risks of several chronic diseases, likely because of the antioxidant and anti-inflammatory activity of caffeine when ingested in infusions [5]. However, the over-consumption (including higher doses) of caffeine can lead to addiction, insomnia, migraine, and other side effects [5]. Additionally, the use of caffeine-containing products in children, adolescents and pregnant women should be restricted as the potential side effects of caffeine are exacerbated in these populations [6]. In sports, there is also a high consumption of caffeine [7,8], because of the wide physical and physiological benefits reported after acute oral caffeine ingestion of doses between 3 and 9 mg/kg of caffeine in individual and team sports [9,10].

Beyond the well-supported effect of oral caffeine intake to enhance some aspects of health and sports performance, this substance can exert other potential benefits such as increased fat utilization at rest and during aerobic exercise [11]. Specifically, it has been shown by studies with a meta-analytical approach that caffeine can have a significant impact on increasing the rate of fat oxidation at rest [11] and during aerobic exercise at low and moderate intensities [7]. Additionally, it has been found that the effect of caffeine to enhance fat oxidation during exercise is similar in the range of 3 to 6 mg per kg of body mass and of similar magnitude in men and women [12,13]. The mechanism by which caffeine increases fat oxidation is through the activation of the sympathetic nervous system, which ultimately increases the release of fatty acids and augments lipolysis [14]. Interestingly, this is a mechanism different to the one reported for the stimulant effects of this substance (i.e., blockade of adenosine receptors [15]) and this likely explains why caffeine would exert a role on fat oxidation during low-to-moderate intensity exercise or at rest. The influence of caffeine to increase fat utilization during exercise may be an attractive effect for those individuals enrolled in programs seeking body weight reduction and fat mass loss. In this context, the caffeine-induced effect in increasing blood pressure and insomnia should also be considered, especially when using caffeine to enhance fat oxidation in obese or overweight individuals who may possess other cardiovascular comorbidities. 

The higher fat oxidation rate at rest and during exercise found after caffeine intake may potentially aid in producing a faster reduction in fat mass for those individuals seeking body fat-mass loss. However, to produce a measurable benefit, this substance should be ingested chronically in conjunction with a weight loss program or an exercise training protocol, as the effect of caffeine to augment fat oxidation is of the order of ~0.08 to 0.20 g/min during exercise [16,17,18]. Despite the mounting evidence suggesting that caffeine may enhance fat oxidation, some experts have suggested that more research with robust methodological designs is needed to determine the long-term effects of caffeine intake on fat mass reduction and body composition change [19]. It is still needed to resolve the practical questions to effectively apply oral caffeine intake to enhance fat oxidation, such as the best source for caffeine administration, the most effective timing of ingestion, and the potential need to combine fasting and caffeine intake to obtain the benefits of this substance on fat oxidation rates at rest and during exercise. An explanation as to why some people are more sensitive to the effects of oral caffeine intake than others is also needed as this may imply the identification of genetic variants that increase/decrease the potential benefits of caffeine [20]. This is important as there are data that indicate that a single nucleotide polymorphism in the *CYP1A2* gene, known as the c.-163A>C (rs762551), modifies the activity of the CYP1A2 enzyme, which is responsible for most of the caffeine metabolism in the liver [21]. 

As it seems necessary to broaden the knowledge related to oral caffeine intake and its effects on fat oxidation, analysis of the previous literature may help understand the evolution of research on this topic to ultimately provide an evolved vision of future research directions pertinent to caffeine use. Hence, the aim of this study was to carry out a bibliometric review in relation to the concepts “caffeine” and “fat oxidation”, to understand the evolution of research related to the effect of oral caffeine intake on the rate of oxidation of fat, from 1992 to 2022.

## 2. Materials and Methods

### 2.1. Study Design

The present study is framed within the area of retrospective studies [22] since events that previously occurred are analyzed. In the same way, it is located within the frame of theoretical studies [23], because a review of the literature is carried out under the premise of bibliometric studies [24].

### 2.2. Data Extraction

For the search and compilation of the documents, the Web of Science (WoS) database was used following prior recommendations [25,26,27], allowing the extraction of relevant information for each published study on the topic such as the title, year of publication, abstract, keywords, author affiliations, type of document, among other information. 

### 2.3. Search Strategy

**Figure 1:**
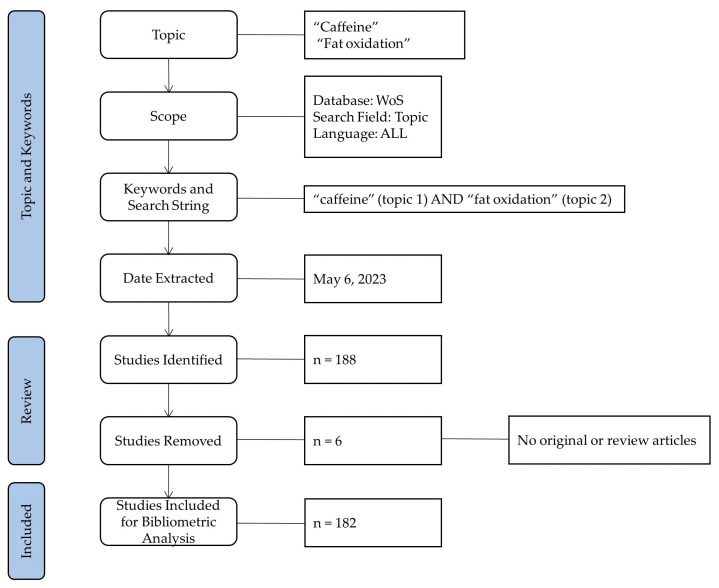
Flux diagram of data collection.

For the search of documents, the following keywords were used: “caffeine” and “fat oxidation”, using the “topic” filter to identify those documents that include these keywords in the title, abstract, or keywords. There was no filter applied to the form of caffeine administration, so the search included studies using “pure” caffeine in capsules/tablets or caffeinated drinks and foodstuffs such as coffee, tea, or energy drinks. The search for the documents was carried out by two researchers (VHB and JMG) on 6 May 2023, and disagreements in the search process and identification of relevant papers were resolved by discussion with a third author (ME). The literature search was conducted from the inception of the database (1992) to 31 December 2022, with no year or language restriction for the search strategy. The initial search retrieved a total of 188 potentially relevant documents. Articles that appeared potentially relevant because of their title were further reviewed by consulting their title and abstract. If an article was deemed eligible based on its title and abstract, the full text was immediately reviewed for further assessment. Abstracts in conferences, books, and book chapters, and documents with early access by the WoS were excluded; so, only original and review articles were included in this analysis [28]. After reviewing the titles and abstracts, six articles were discarded and a total of one hundred and eighty-two valid research documents were used for the bibliometric analysis. The selection of the articles was carried out by two researchers (VHB and JMG). Figure 1 shows the details of the search and study selection methodology.

### 2.4. Data Analysis

For the statistical analysis, the most relevant laws of bibliometric reviews were considered [24]. To evaluate the exponential growth of the selected documents, Price’s law [29] was used, through the calculation of the coefficient R^2^. In this way, the trend in the increase in the number of documents published in relation to the topic was deduced. In addition, two time-zones were established to categorize the documents in terms of year of publication: older documents, as the documents published before the median of the sample, which was 2009, and newer manuscripts, for those documents published in or after 2010. To identify those authors with the largest output of published research documents on the topic, the Lotka analysis [30] was carried out, extracting the H-Index for each author identified in the search [31]. All the authors of each study were extracted and analyzed, independently of the position they had in the list of authors consigned in the research document. The H-index was defined as the maximum value of h, such that the given author has published at least h papers that have each been cited at least h times [32]. The H-Index was employed to identify those authors with the potential greatest contribution to the field under scrutiny [33]. Additionally, the affiliations of each author were also collected and meta-analyzed to detect organizations with the highest research output in the topic. 

In addition, to analyze the keywords most used by the authors in each of the papers (*n* = 372), Zipf’s law was used [34,35]. Finally, for data analysis and visualization, Microsoft Excel (2006 version: Microsoft Corporation, Redmond, WA, USA) and VOSviewer (v.1.6.19 for macOS, Center for Science and Technology Studies, Leiden, The Netherlands) were used. For the creation and visualization of the results, a fragmentation analysis was used (attraction: 3 and repulsion: −3), depending on the theme and the temporality of the results [36].

## 3. Results

### 3.1. Evolution in the Number of Publications

**Figure 2:**
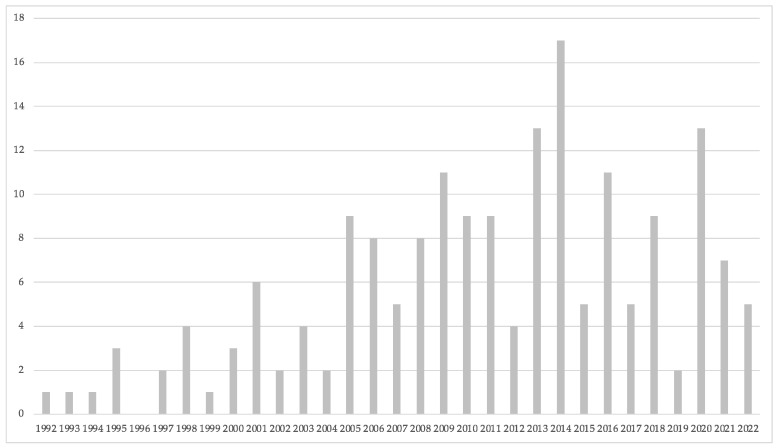
Yearly evolution of the number of scientific articles published on the effect of caffeine intake on fat oxidation.

Figure 2 shows the evolution in the number of publications across the years. A total of 182 documents were identified after the search between 1992 and 2022, of which 157 were scientific and original articles, and 25 were review articles. The first document related to the topic was published in 1992. A continuation in the periodicity of the publications was not observed because no published documents were identified in 1996. Therefore, the exponential growth was analyzed between 1997 and 2022. The R^2^ coefficient shows an exponential growth of 20.1% comparing the number of publications between the years 1997 and 2009 (*n* = 73) and 2009 and 2022 (*n* = 109). It can be observed that the year with the highest number of publications was 2014 (*n* = 17), with a progressively lower number of articles afterwards. 

### 3.2. Web of Science Categories

Table 1 shows the number of articles according to the categories established by the WoS. This database categorizes articles according to the main topic developed in the study; however, a document can belong to two different categories, as multidisciplinary themes or issues can be developed. For this, the first 10 disciplines were selected, as well as the number of manuscripts assigned to these categories. The percentage of articles within each category represents the proportion of such category with respect to the total number of articles published. Overall, the category of “Nutrition & Dietetics” included more than half of the documents published in the topic (*n* = 108), followed by “Sport Science” (*n* = 48), totaling between both categories 85.71% of the documents published about caffeine and fat oxidation.

### 3.3. Most Cited Documents

**Figure 3:**
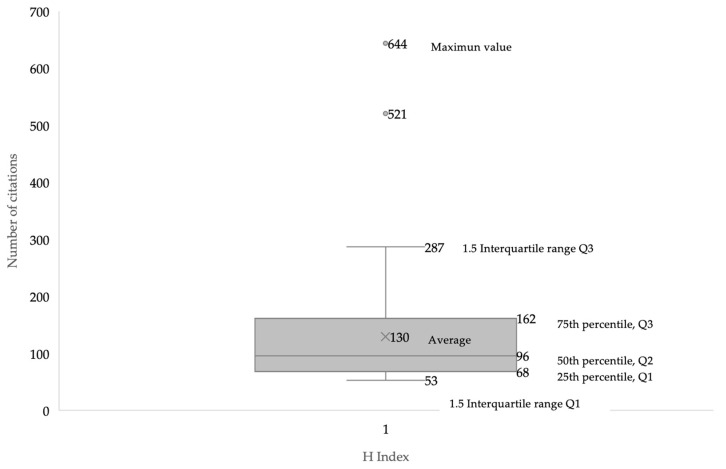
Mean spread of citations of published articles associated with the effect of caffeine intake on fat oxidation.

Figure 3 shows data about the number of citations received by the documents included in the search. There are two documents with a much higher number of citations than the remaining documents: the study by Dulloo et al. [37], with 644 citations, and the study by Graham [38], with 521 citations. Overall, the average number of citations per document is 130. Additionally, a total of 52 authors with a minimum of 52 citations have been identified which represents an H index for the topic of 52.

Next, Table 2 shows the most cited documents regarding the number of total citations, and the average number of citations per year. The paper by Dulloo et al. [36] is the most relevant research document taking into account its ~26 citations per year and it is followed by the document published by Sinha et al. [39], with 23 citations per year.

### 3.4. Scientific Journals

Table 3 shows the top ten scientific journals with more documents published on the topic, along with their 2022 impact factor. The journal “Nutrients” has published 12 documents followed by the “British Journal of Nutrition” with 10 documents. Overall, the publication of documents is dispersed in terms of journals as there are no journals with a quota higher than 10% of the total number of articles published.

### 3.5. Publications Considering the Countries

**Figure 4:**
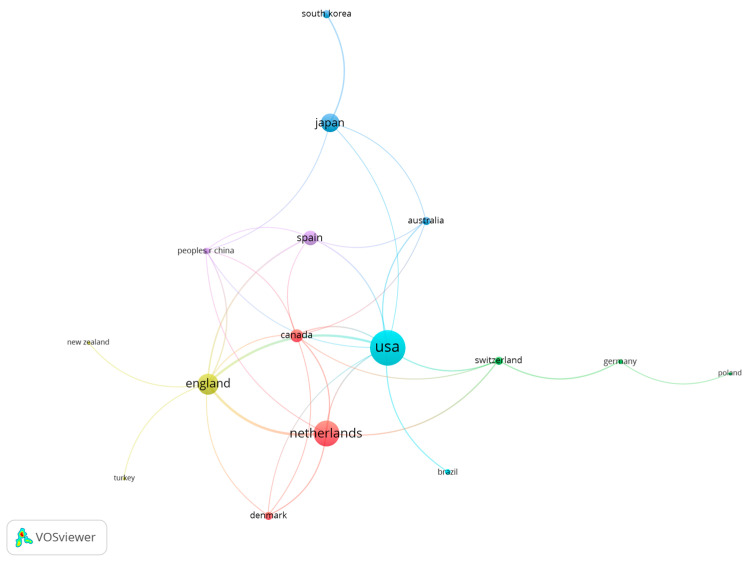
List of countries based on the number of documents produced.

**Figure 5:**
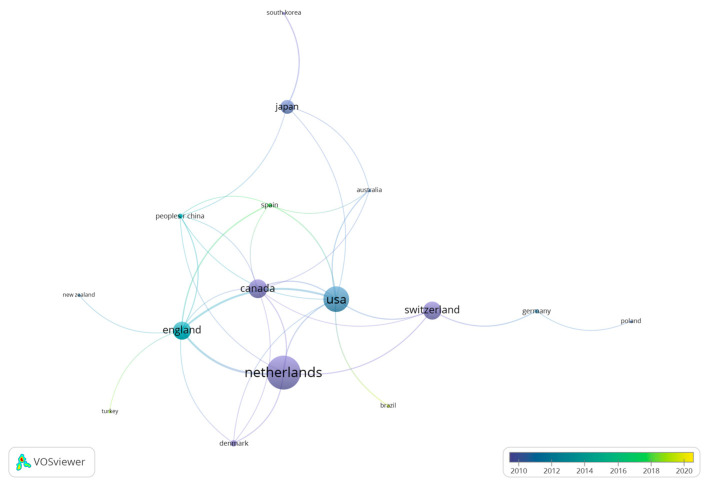
List of countries based on temporality and the number of received citations.

The search depicted that authors from a total of 30 countries have published documents about caffeine and fat oxidation. Seventeen countries presented a minimum of three research documents, and fourteen countries had a minimum of four published documents. Figure 4 shows the relationship among the different countries or regions included in the analysis, including those countries with a minimum of three documents. Overall, the United States is the country with the highest number of publications (*n* = 44), followed by the Netherlands (*n* = 31), and England (*n* = 25). On the contrary, taking the number of citations received as a reference, the Netherlands is the country with the highest number of citations (*n* = 2456), followed by the United States (*n* = 1854; Figure 5).

### 3.6. Network between Authors

Overall, there were 752 authors with a contribution to the topic with a distribution of between 1 and 7 authors per document. There were 103 authors who published a minimum of 2 documents, and 33 authors who had at least 3 documents published. Table 3 lists the 10 most relevant authors on the topic, regardless of their position in the list of authors of the published documents. Westerterp-Platenga, Jeukendrup, and Hursel were the authors with the highest number of published documents with 16 and 11 documents, respectively. Table 4 also includes the H Index of the most productive authors on the topic selected with Westerterp-Platenga and Jeukendrup also obtaining the highest H Index values.

**Figure 6:**
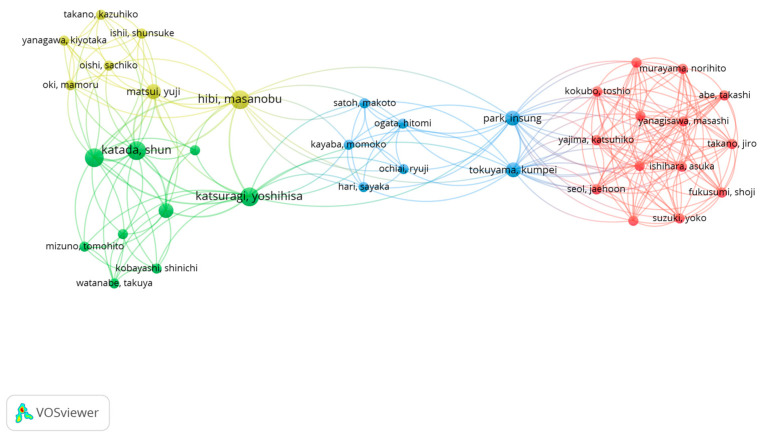
Main collaborative networks between authors linked to the effect of caffeine intake on fat oxidation.

Including the 752 authors in the analysis shows the existence of 4 main collaborative networks between the authors, and 37 co-authorship networks (Figure 6). It shows that the main collaborative networks linked to the effect of caffeine intake on fat oxidation are between authors from Japan.

### 3.7. Publications Regarding Organizations

**Figure 7:**
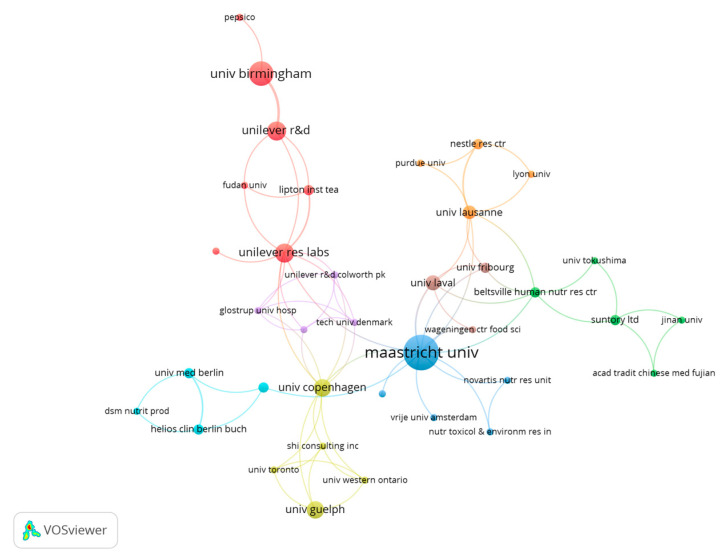
Main collaborative networks among institutions publishing research documents on the effect of caffeine on fat oxidation.

A total of 260 organizations were identified as affiliations of the 182 documents selected. From this total, only 10 institutions have at least 5 documents published, and only 3 organizations have published ≥ 7 documents. Figure 7 shows the relationship between the different organizations with documents published on the effect of caffeine on fat oxidation. The University of Maastricht has the highest number of documents (*n* = 21), followed by the University of Birmingham (*n* = 10). 

### 3.8. Keywords Used by Authors

**Figure 8:**
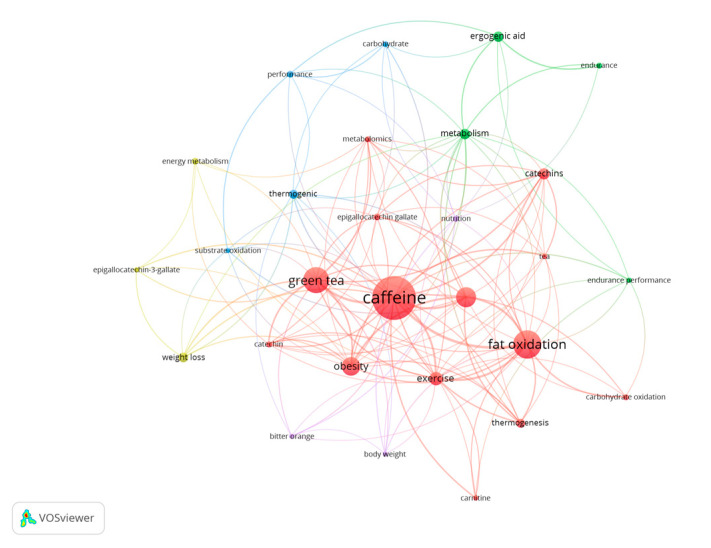
Network of keywords used in research documents on the effect of caffeine on fat oxidation.

A total of 372 keywords have been identified in the different studies with 28 keywords included in more than five documents. The terms that present the highest frequency of use in the documents are “caffeine” (*n* = 50), “fat oxidation” (*n* = 31), “green tea” (*n* = 29), “energy expenditure” (*n* = 22), and “obesity” (*n* = 20) (Figure 8).

**Figure 9:**
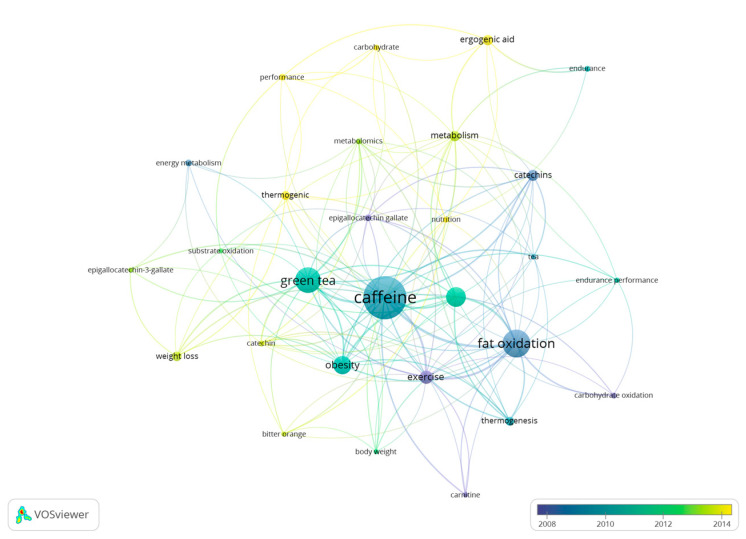
List of keywords based on the year of publication.

**Figure 10:**
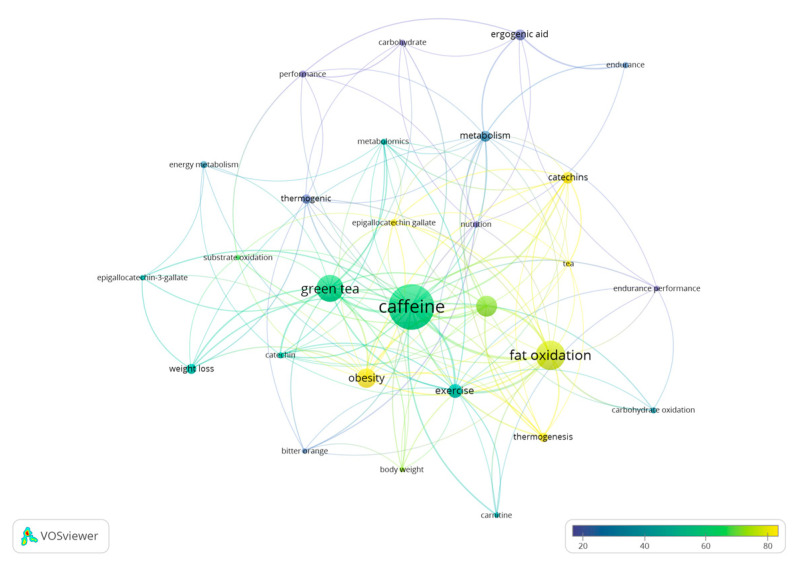
List of terms based on the average number of citations received.

Assuming the temporality changes in the keywords as a reference, a tendency is present to include keywords such as “performance”, “carbohydrate”, and “ergogenic aid” in the newer articles (Figure 9). Therefore, due to their recent use and inclusion in newer research documents, these terms are the ones that present a lower number of citations. On the other hand, “obesity”, “thermogenic”, “tea”, or “catechin” are the keywords with the highest number of citations (Figure 10), as they are keywords more commonly included in older documents. 

## 4. Discussion

The aim of this study was to assess the research activity regarding oral caffeine intake and fat oxidation rate in the last few decades by conducting a bibliometric and visual analysis with the concepts “caffeine” and “fat oxidation”. The final goal of this review is to evaluate the evolution of research related to the effect of oral intake of caffeine on fat oxidation from 1992 to 2022 to understand the most recurrent themes of research on this topic and to help direct future research. The outcomes reported by this analysis may be used by researchers to assess the current scientific interest in the use of caffeine as a nutritional strategy to augment fat oxidation, the journals with a greater interest in this type of publication, and to draw international collaborations between groups working in the same area. The search revealed the existence of 182 documents published about caffeine and fat oxidation with a 20.1% growth in the number of publications between the years 1997 and 2009 (*n* = 73) and 2009 and 2022 (*n* = 109). The main journals of publication were Nutrients (*n* = 12), and the British Journal of Nutrition (*n* = 10), as most of the documents were included in the WoS category of nutrition and dietetics. Although the main collaborative networks linked to the effect of caffeine intake on fat oxidation are between authors from Japan, the most productive countries in the number of documents and citations received were the United States and the Netherlands. Last, a tendency to use keywords more associated with the use of caffeine in the exercise performance context and less in the obesity context is appreciated in the newer documents. Carrying out a bibliometric analysis is essential to determine qualitative and quantitative trends in each research topic [47] and provides useful information for experts seeking to evaluate the scientific activity and future directions [48]. Future investigations may focus on the effects of sex and tolerance to caffeine to widen the assessment of the effectiveness of oral caffeine intake as a nutritional strategy to augment the use of fat as a fuel, as these themes were rarely included in the documents retrieved in the search.

From the total 182 documents of the search, 157 were original articles and 25 were review articles, showing that this topic contains a high proportion of studies with novel data. Regarding the number of documents based on the categories established by the WoS platform, the first category is nutrition and dietetics (*n* = 108), and the second category is sport sciences (*n* = 48). Both categories encompass 85.71% of the manuscripts related to the evolution of research related to the effect of caffeine intake on fat oxidation, from its origin to the year 2022. From a practical perspective, these data indicate that the research on this topic should be prepared to be understood by potential readers with a mixed knowledge of nutrition and sports sciences. This is important as these professionals would be the main facilitators for translating the research findings associated with oral caffeine intake at rest and during exercise to science-based protocols in real-life scenarios. To be successful in the translational approach of this topic to the real world, it is indispensable that the research papers are written and focused on being understood by this segment of the population; this focus should be considered for future documents published in this topic with the final aim of facilitating the application of research results. Interestingly, Figure 2 shows that there is no exponential growth year after year despite the progressively higher number of journals included in the WoS. In the scientific literature, there are no other bibliometric studies that corroborate the results obtained; however, these data indicate that the scientific interest in the role of caffeine as an agent to modify fat oxidation at rest or during exercise has experienced little change over the years. Overall, the year with the highest number of publications is 2014, and a tendency for a lower number of papers published since then is present, suggesting that this topic is now receiving less scientific attention than a decade ago. These data contrast with the relatively high number of citations per study (i.e., 130), suggesting that there may be a steady-state activity for publishing new studies on the effect of caffeine on fat oxidation due to the difficulty of performing experiments with supplementation protocols in humans, while the scientific interest of the research papers published is still high. Although it is not the objective of this analysis, this may be the result of the low recompense that exists, in terms of costs/benefits, when publishing studies with original data in humans, at least for some topics. Another possible explanation for the lack of a progressive increase in the number of publications per year may indicate a possible saturation of scientific interest in this topic.

Bibliometric reviews present unique opportunities to contribute to theory and practice. However, studies using bibliometric techniques have often attracted criticism for failing to adequately link their derived analytic and visual results to theory-building and practice improvement [49]. We collectively believe that the present study offers useful information for researchers as it shows the evolution in the number of papers published over the years, the average number of citations received per document, the journals that have accepted more documents, and the most productive authors, organizations, and worldwide collaborative networks. With this information, authors studying the role of caffeine on substrate oxidation at rest and during exercise may gauge the impact that their research may have in the field and the journals and authors that may be potentially more interested in reading/reviewing their research. Additionally, Figure 8, Figure 9 and Figure 10 show the keywords more recurrently employed in the papers, in general, and with respect to the year of publication, which also may help authors to focus on themes more actual or with the greatest interest.

The results related to the H Index of the selected manuscripts show the existence of 52 authors with a minimum of 52 citations. The studies of Dulloo et al. [37], with 644 citations, and by Graham [38], with 521 citations were the most cited documents in the scientific literature related to the study topic. These data show that the most cited documents in the topic are older than 20 years and only the study by Sinha et al. is within the top ten most cited documents with less than 10 years since the date of publication (i.e., 2014). Considering the results related to co-authorship, the most relevant researchers were Westerterp-Platenga, Jeukendrup and Hursel. Likewise, there are four main collaboration networks between the authors, and thirty-seven co-authorship networks. Concerning the publications by country, the United States is the country with the highest number of publications (*n* = 44), followed by the Netherlands (*n* = 31), and England (*n* = 25). Regarding the number of citations received, the Netherlands is the country with more citations obtained (*n* = 2456), followed by the United States (*n* = 1854). The University of Maastricht and the University of Birmingham are the organizations with more publications on the topic. In the scientific literature, there are no previous analysis to corroborate the data obtained, since no bibliometric review related to the object of study has been carried out previously. However, this information may be interesting for researchers on caffeine and fat oxidation to establish future lines of work [47], as well as the knowledge of research groups and institutions with a high number of publications and citations may enhance the quality of future research. 

The results related to the keywords employed in the documents show the existence of 372 different terms identified with a higher frequency of use of “caffeine” (*n* = 50), “fat oxidation” (*n* = 31), “green tea” (*n* = 29), “energy expenditure” (*n* = 22), and “obesity” (*n* = 20). Regarding the temporality of the terms, there is a change in the trend, and the most recent keywords are “performance”, “carbohydrate”, and “ergogenic aid”. The results show the existence of an evolution in terms of the terms used by researchers, likely associated with the use of caffeine in the sporting context. Although future bibliometric studies must confirm this tendency, this may indicate a progressively higher scientific interest in the use of caffeine to modify fat oxidation during exercise in athletes and recreationally active individuals and a progressively lower interest in the use of this substance in disease populations such obese individuals. 

## 5. Conclusions

The results of this bibliometric review related to caffeine intake and its effects on fat oxidation at rest and during exercise show the existence of 182 documents (157 scientific articles and 25 review articles) published between 1992 and 2022. It seems that this topic may have reached its peak, as the year with the highest number of documents was 2014 and there has been a lower number of publications per year since then, possibly indicating saturation. However, this topic is still a subject of interest for the scientific community in nutrition and exercise physiology, as the average number of citations received is 130 and there are 52 authors with at least 52 citations. These documents were mainly published in the WoS categories of nutrition and dietetics and sport sciences with four main collaboration networks between different authors in Japan. Lastly, there is a higher progressive interest in the use of caffeine to augment fat oxidation in the sporting context reflected by the higher use of keywords such as ergogenic aid and performance. Future investigation may focus on the effects of sex and tolerance to caffeine to widen the assessment of the effectiveness of oral caffeine intake as a nutritional strategy to augment the use of fat as a fuel, as these keywords rarely appeared in the documents identified in the search.

## Figures and Tables

**Table 1:** Distribution of documents on the effect of caffeine on fat oxidation according to the categories established by WoS.

Category	*n*	*%*
Nutrition and Dietetics	108	59.34
Sport Sciences	48	26.37
Endocrinology and Metabolism	25	13.74
Physiology	19	10.44
Food Science and Technology	12	6.59
Biochemistry and Molecular Biology	8	4.40
Behavioral Sciences	5	2.75
Medicine, Research and Experimental	5	2.75
Multidisciplinary Sciences	5	2.75
Agriculture, Multidisciplinary	4	2.20

**Table 2:** Ten most-cited research documents associated with the effect of caffeine intake on fat oxidation.

Title	Authors	Journal Title	Publication Year	Total Citations	Average Citations per Year
Efficacy of a green tea extract rich in catechin polyphenols and caffeine in increasing 24-h energy expenditure and fat oxidation in humans	Dulloo et al. [37]	American Journal of Clinical Nutrition	1999	644	25.76
Caffeine and exercise—Metabolism, endurance and performance	Graham [38]	Sports Medicine	2001	521	22.65
Antiobesity effects of green tea catechins: a mechanistic review	Rains et al. [40]	Journal of Nutritional Biochemistry	2011	287	22.08
Caffeine Stimulates Hepatic Lipid Metabolism by the Autophagy-Lysosomal Pathway in Mice	Sinha et al. [39]	Hepatology	2014	230	23.00
Body weight loss and weight maintenance in relation to habitual caffeine intake and green tea supplementation	Westerterp-Plantenga et al. [41]	Obesity Research	2005	224	11.79
Anti-obesity effects of three major components of green tea, catechins, caffeine and theanine, in mice	Zheng et al. [42]	In Vivo	2004	220	11.00
The effects of green tea on weight loss and weight maintenance: a meta-analysis	Hursel et al. [43]	International Journal of Obesity	2009	205	13.67
The Metabolic and Performance Effects of Caffeine Compared to Coffee during Endurance Exercise	Hodgson et al. [44]	PLoS ONE	2013	204	18.55
Green tea extract ingestion, fat oxidation, and glucose tolerance in healthy humans	Venables et al. [45]	American Journal of Clinical Nutrition	2008	197	12.31
Effect of caffeinated drinks on substrate metabolism, caffeine excretion, and performance	Kovacs et al. [46]	Journal of Applied Physiology	1998	178	6.85

Note: The number of citations includes self-citations as they cannot be removed in the analysis of the Web of Science.

**Table 3:** Scientific journals with more documents published on the effect of caffeine intake on fat oxidation.

Journal	*n*	%	IF *
Nutrients	12	6.59	5.9
British Journal of Nutrition	10	5.49	3.6
Medicine and Science in Sports and Exercise	9	4.94	5.4
American Journal of Clinical Nutrition	8	4.39	7.1
Journal of The International Society of Sports Nutrition	8	4.39	5.1
Journal of Nutritional Science and Vitaminology	7	3.84	1.6
European Journal of Nutrition	6	3.29	5.0
International Journal of Obesity	5	2.74	4.9
International Journal of Sport Nutrition and Exercise Metabolism	5	2.74	2.5
Applied Physiology Nutrition and Metabolism	4	2.19	3.4

* Impact Factor in the 2022 Journal Citation Reports.

**Table 4:** Most relevant authors related to the effect of caffeine intake on fat oxidation.

Author	*n*	*%*	*H Index*
Westerterp-Plantenga, M.S.	16	8.79	65
Jeukendrup, A.E.	11	6.04	72
Hursel, R.	10	5.49	17
Del Coso, J.	9	4.95	37
Lim, K.	9	4.95	23
Kovacs, E.M.	8	4.40	25
Mela, D.J.	8	4.40	41
Hodgson, A.B.	7	3.85	10
Randell, R.K.	7	3.85	13
Gutiérrez-Hellín, J.	6	3.30	11

## Data Availability

The data that support the findings of this study are available from the corresponding author, upon reasonable request.
